# A chromosome-level, fully phased genome assembly of the oat crown rust fungus *Puccinia coronata* f. sp. *avenae*: a resource to enable comparative genomics in the cereal rusts

**DOI:** 10.1093/g3journal/jkac149

**Published:** 2022-06-22

**Authors:** Eva C Henningsen, Tim Hewitt, Sheshanka Dugyala, Eric S Nazareno, Erin Gilbert, Feng Li, Shahryar F Kianian, Brian J Steffenson, Peter N Dodds, Jana Sperschneider, Melania Figueroa

**Affiliations:** Department of Plant Pathology, University of Minnesota, St. Paul, MN 55108, USA; Commonwealth Scientific and Industrial Research Organisation, Agriculture and Food, Canberra, ACT 2601, Australia; Present address: Research School of Biology, The Australian National University, Canberra, ACT 2601, Australia; Commonwealth Scientific and Industrial Research Organisation, Agriculture and Food, Canberra, ACT 2601, Australia; Department of Plant Pathology, University of Minnesota, St. Paul, MN 55108, USA; Department of Plant Pathology, University of Minnesota, St. Paul, MN 55108, USA; Bayer CropScience, Creve Coeur, MO 63141, USA; eGenesis Inc., Cambridge, MA 02139, USA; Department of Plant Pathology, University of Minnesota, St. Paul, MN 55108, USA; USDA-ARS Cereal Disease Laboratory, St. Paul, MN 55108, USA; Department of Plant Pathology, University of Minnesota, St. Paul, MN 55108, USA; Commonwealth Scientific and Industrial Research Organisation, Agriculture and Food, Canberra, ACT 2601, Australia; Commonwealth Scientific and Industrial Research Organisation, Agriculture and Food, Canberra, ACT 2601, Australia; Commonwealth Scientific and Industrial Research Organisation, Agriculture and Food, Canberra, ACT 2601, Australia

**Keywords:** oat, rust, virulence, genome, chromosome, isolate *Pca*203

## Abstract

Advances in sequencing technologies as well as development of algorithms and workflows have made it possible to generate fully phased genome references for organisms with nonhaploid genomes such as dikaryotic rust fungi. To enable discovery of pathogen effectors and further our understanding of virulence evolution, we generated a chromosome-scale assembly for each of the 2 nuclear genomes of the oat crown rust pathogen, *Puccinia coronata* f. sp. *avenae* (*Pca*)*.* This resource complements 2 previously released partially phased genome references of *Pca*, which display virulence traits absent in the isolate of historic race 203 (isolate *Pca*203) which was selected for this genome project. A fully phased, chromosome-level reference for *Pca*203 was generated using PacBio reads and Hi-C data and a recently developed pipeline named NuclearPhaser for phase assignment of contigs and phase switch correction. With 18 chromosomes in each haplotype and a total size of 208.10 Mbp, *Pca*203 has the same number of chromosomes as other cereal rust fungi such as *Puccinia graminis* f. sp. *tritici* and *Puccinia triticina*, the causal agents of wheat stem rust and wheat leaf rust, respectively. The *Pca*203 reference marks the third fully phased chromosome-level assembly of a cereal rust to date. Here, we demonstrate that the chromosomes of these 3 *Puccinia* species are syntenous and that chromosomal size variations are primarily due to differences in repeat element content.

## Introduction

The rust fungus *Puccinia coronata* f. sp. *avenae* (*Pca*) is the most damaging foliar pathogen of oat (*Avena sativa* L.) (Nazareno *et al.* 2018). *Pca* is commonly found in areas of the world where oats are grown and can destroy up to 50% of the crop during severe epidemics (USDA-ARS CDL 2014; Nazareno *et al.* 2018). Plants have race-specific resistance (*R*) genes, which generally encode immunoreceptors that recognize pathogen effectors as a strategy to stop infection and disease development (Dodds and Rathjen 2010). The molecular basis of virulence in *Pca* is not well characterized and this is partly due to the lack resources to direct such studies. High quality genome references of the pathogen are instrumental to investigate the underlying mechanisms of virulence, enable effector discovery, and even accelerate the identification of *R* genes (Figueroa *et al.* 2016, 2020; Upadhyaya *et al.* 2021).

The dikaryotic nature of rust fungi means their genetic information is present in 2 haplotypes that are physically separated in 2 nuclei for most of their life cycle. This feature in combination with highly repetitive genomes poses some technical challenges for genome assembly. Until recently, genome references built for rust fungi did not fully capture sequence information from both nuclei and most assemblies were haploid representations of the genome with abundant haplotype sequence (phase) swaps (Cantu *et al.* 2011; Cuomo *et al.* 2017). New technologies such as long-read and Hi-C sequencing (Li *et al.* 2019) as well as the recent development of the NuclearPhaser pipeline (Duan *et al.* 2022) have improved our ability to assemble chromosome references and fully resolve the 2 haplotypes in rust fungi. To date the only existing chromosome-level nuclear phased assemblies belong to *Puccinia graminis* f. sp. *tritici* and *Puccinia triticina*, which cause stem and leaf rust on wheat, respectively. There are 2 *Pca* genome assemblies (Miller *et al.* 2018), which were the first attempt toward haplotype resolution of any rust fungi and consist of a primary pseudo-haplotype assembly, with a partially resolved secondary haplotype that is only 50–60% complete.

Here, we selected another *Pca* isolate, *Pca*203, for assembly of a complete haplotype-phased genome reference using state-of-art de novo genome assembly approaches for dikaryotic rust fungi. *Pca*203 represents an isolate of the historic *Pca* race 203, which caused severe epidemics in the early 1940s in the USA (Stoa and Swallers 1950) leading to the deployment of the *Pc2* resistance gene present in Victoria oats. The subsequent widespread cultivation of Victoria oats led to devastating epidemics of *Cochliobolus victoriae*, likely as a result of *Pc2* acting as a susceptibility factor for the Victorin toxin produced by this necrotrophic pathogen (Murphy and Meehan 1946; Wolpert *et al.* 2002). Thus, a gold-standard assembly of *Pca*203 could bring insights into one of the best-known classical problems of plant pathology. Furthermore, the contrasting virulence profile of *Pca*203 and other previously sequenced *Pca* isolates will help unravel the molecular basis of *Pca* virulence and aid in future comparative genomic studies within *Pca* and other rust species.

## Materials and methods

### Plant and fungal materials and plant inoculations

An isolate of *Pca* race 203, known to be avirulent to the oat cultivar Victoria (Chang and Sadanaga 1964), was retrieved from storage at the USDA-ARS Cereal Disease Laboratory (CDL), Saint Paul, MN, USA (Omidvar *et al.* 2018). From this culture, a single pustule was isolated and increased to ensure sample purity. The virulence phenotype of *Pca*203 was determined according to a current standard nomenclature system using a set of oat differentials (Chong *et al.* 2000; Nazareno *et al.* 2018). Urediniospore stocks were kept at −80°C. As previously described by Miller *et al.* (2018), virulence phenotypes of *Pca* on the oat differentials were converted to a 0–9 numerical scale for heat map generation using R packages ComplexHeatmap and circlize (Gu *et al.* 2016, 2014). Seed from the oat differential set was also obtained from the USDA-ARS CDL. Urediniospores increases were completed on the oat cv. Marvelous as a susceptible host. Oat inoculations with *Pca* were carried out as described by Omidvar *et al.* (2018).

### DNA and RNA isolation and sequencing

High molecular weight DNA was extracted from 700 mg of urediniospores, as previously described by Li *et al.* (2019) and sent to the University of Minnesota Genomics Center (UMGC, St. Paul, MN, USA) for library construction using the PacBio SMRTbell 1.0 kit and sequencing using 5 PacBio Sequel System SMRT cells with v3 chemistry. DNA was also extracted from 20 mg of isolate *Pca*203 spores using the Omniprep DNA isolation kit from G-Biosciences for library preparation with the Illumina TruSeq Nano DNA protocol and Illumina NovaSeq sequencing in the S2 flow cell at UMGC to produce 150 bp paired-end reads. For DNA-crosslinking and Hi-C sequencing, 100 mg of spores was suspended in 15 ml 1% formaldehyde and incubated at room temperature (RT) for 20 min with periodic vortexing. Glycine was added to 1 g/100 ml, and the suspension was incubated again at RT for 20 min with periodic vortexing. The suspension was centrifuged at 1,000 *g* for 1 min and the supernatant was removed; the spores were then transferred to a liquid nitrogen-cooled mortar and ground before being stored at −80°C or on dry ice. After crosslinking, treated spores were sent to Phase Genomics (Seattle, WA, U.S.A) for DNA extraction and Hi-C library preparation with the Proximo Fungal 4.0 protocol and libraries were sequenced to 100 million 150 bp paired-end reads at Genewiz (South Plainfield, NJ, USA). RNA was extracted from infected oat cv. Marvelous at 2- and 5-days post inoculation (dpi) using the Qiagen RNeasy Plant Mini Kit following the manufacturer’s instructions and sent to UMGC for library preparation with the Illumina TruSeq Stranded mRNA protocol and sequencing using Illumina NextSeq in mid-output mode, producing 75 bp paired-end reads.

### Genome assembly and polishing

Rust isolate purity was assessed by examining SNP allele balance as previously described (Miller *et al.* 2018). For this, Illumina short reads were trimmed with trimmomatic version 0.33 (Bolger *et al.* 2014) and aligned with bwa version 0.7.17 (Li and Durbin 2009) to the existing 12SD80 primary genome assembly (Miller *et al.* 2018). The alignments were processed using samtools version 1.9 (Li *et al.* 2009), and variants were called with Freebayes version 1.1.0 (Garrison and Marth 2012). The allele balance plot was generated using a custom R script (https://github.com/henni164/Pca203_assembly/blob/master/figure_s2/203_frequencies.R).

The genome was assembled using Canu version 2.1 with setting genomeSize = 200m (Koren *et al.* 2017). For polishing with the PacBio read data, half of the subreads were mapped back to the assembly using PacBio software pbmm2 version 1.4.0 (https://github.com/PacificBiosciences/pbmm2/releases/tag/v1.4.0) and a new consensus generated with PacBio software GenomicConsensus version 2.3.3. This process was repeated using the new consensus as the reference. Next, the updated consensus was polished twice in Pilon version 1.22 (–fix indels) using Illumina short reads trimmed with trimmomatic version 0.33 (Bolger *et al.* 2014; Walker *et al.* 2014). Assembly statistics were calculated using Quast version 5.1.0 (Gurevich *et al.* 2013).

### Identification of mitochondrial contigs and removal of contaminants

All contigs were first screened by BLAST against the mitochondrial genome database from NCBI with ncbiblast+ version 2.8.1, and mitochondrial contigs were removed from the main assembly (Camacho *et al.* 2009). The remaining contigs were screened against the NCBI nucleotide library and 8 other likely contaminant contigs were removed. Finally, 8 contigs with PacBio reads coverage <2× were removed. Telomeres and collapsed regions were identified with custom scripts (https://github.com/JanaSperschneider/GenomeAssemblyTools, https://github.com/JanaSperschneider/FindTelomeres). One contig consisting entirely of telomeric repeat reads was also removed.

### Haplotype-phasing and annotation of genome assembly

The NuclearPhaser pipeline was used to correct phase swaps and assign haplotypes as described by Duan *et al.* (2022), except that 2 rounds of phase swap correction instead of one round were conducted. Likely phase swap locations were located by plotting the proportion of Hi-C *trans*- contacts to the partially phased haplotypes, as described in https://github.com/JanaSperschneider/NuclearPhaser. Phase swap breakpoints were identified by examining short read alignments in Integrative Genomics Viewer and choosing positions with either high coverage of multimapping reads (representing highly similar regions which were phased, but assembled into the wrong haplotype) or high coverage of unique mapping reads with high SNP density (representing collapsed regions). Collapsed regions were included in both haplotypes.

For scaffolding of the phased haplotypes, the Hi-C reads were mapped to each haplotype using BWA-MEM version 0.7.17 (Li and Durbin 2009) and alignments were then processed with the Arima Genomics pipeline (https://github.com/ArimaGenomics/mapping_pipeline/blob/master/01_mapping_arima.sh). Scaffolding was performed using SALSA version 2.2 (Ghurye *et al.* 2017, 2019).

De novo repeats were predicted with RepeatModeler 2.0.0 and the option -LTRStruct (Flynn *et al.* 2020). The predicted repeats were merged with the RepeatMasker repeat library and RepeatMasker 4.1.0 was run with this combined repeat database (http://www.repeatmasker.org). The resulting repeat-masked genome was used for gene annotation. RNAseq reads were cleaned with fastp 0.19.6 using default parameters (Chen *et al.* 2018). RNAseq reads were aligned to the genome with HISAT2 (version 2.1.0 –max-intronlen 3000—dta) (Kim *et al.* 2019). Genome-guided Trinity (version 2.8.4 –jaccard_clip –genome_guided_bam—genome_guided_max_intron 3000) was used to assemble transcripts (Grabherr *et al.* 2011). Annotation was performed with funannotate version 1.7.4 (https://github.com/nextgenusfs/funannotate). First, funannotate train was run with the Trinity transcripts. Second, funannotate predict was run on the repeat-masked genome with options –ploidy 2 –optimize_augustus. Third, funannotate update was run (–jaccard_clip). BUSCO scores were obtained by running BUSCO version 3.1.0 (Waterhouse *et al.* 2018). Secreted proteins were predicted with SignalP 4.1 (-t euk -u 0.34 -U 0.34) (Petersen *et al.* 2011) and TMHMM version 2.0 (Krogh *et al.* 2001). A fungal protein was called secreted if it was predicted to have a signal peptide and has no transmembrane domains. Effector proteins were predicted with EffectorP version 3.0 (Sperschneider and Dodds 2021).

Hi-C contact maps were produced with HiC-Pro 2.11.1 (MAPQ = 10) (Servant *et al.* 2015) and Hicexplorer 3.6 (Ramírez et al. 2018; Winter et al. 2018; Wolff et al. 2018, 2020). The normalized Hi-C contact maps were used to plot the distribution of Hi-C links within and between the 2 haplotypes.

### Comparative genomics and phylogenetic analysis

The *Pca*203 genome was aligned to the *Pgt*21-0 and *Pt*76 genomes using D-Genies with the minimap2 alignment setting (Cabanettes and Klopp 2018; Li 2018; Duan *et al.* 2022). Phylogenetic analyses were performed on a set of 63 isolates: 30 isolates from 1990, 30 isolates from 2015 as published by Miller et al. (2020), and the 3 genome reference representatives (12SD80, 12NC29, and *Pca*203). Trimmed Illumina reads were aligned to the 12SD80 reference primary contigs using bwa version 0.7.17 (Li and Durbin 2009). The resulting BAM files were processed with Samtools version 1.9 (Li *et al.* 2009) including removal of duplicate reads and filtering for mapping quality of 30. Variant calling against the 12SD80 reference primary contigs was performed using Freebayes version 1.3.2. The resulting VCF file was filtered with vcffilter in vcflib version 1.0.1 (https://github.com/vcflib/vcflib) using the parameters “QUAL > 20 & QUAL/AO > 10 & SAF > 0 & SAR > 0 & RPR > 1 & RPL > 1 & AC > 0.” Additional filtering for 90% genotyping frequency (<10% missing data), 5% minor allele frequency and for bi-allelic SNPs was performed using vcftools version 0.1.16 (Danecek *et al.* 2011) to give a final VCF file representing 974,924 variant sites. To analyze reticulation in *Pca*, an unrooted phylogenetic network was created using SplitsTree version 4.16.2 (Huson and Bryant 2006). The network tree was exported in scalable vector graphics format and labels modified using Inkscape version 1.1.1 (https://inkscape.org/). Orthogroups were predicted with Orthofinder 2.5.4 (Emms and Kelly 2019).

## Results

### Virulence profile and pathotyping of *Pca* isolate 203

**Fig. 1.**
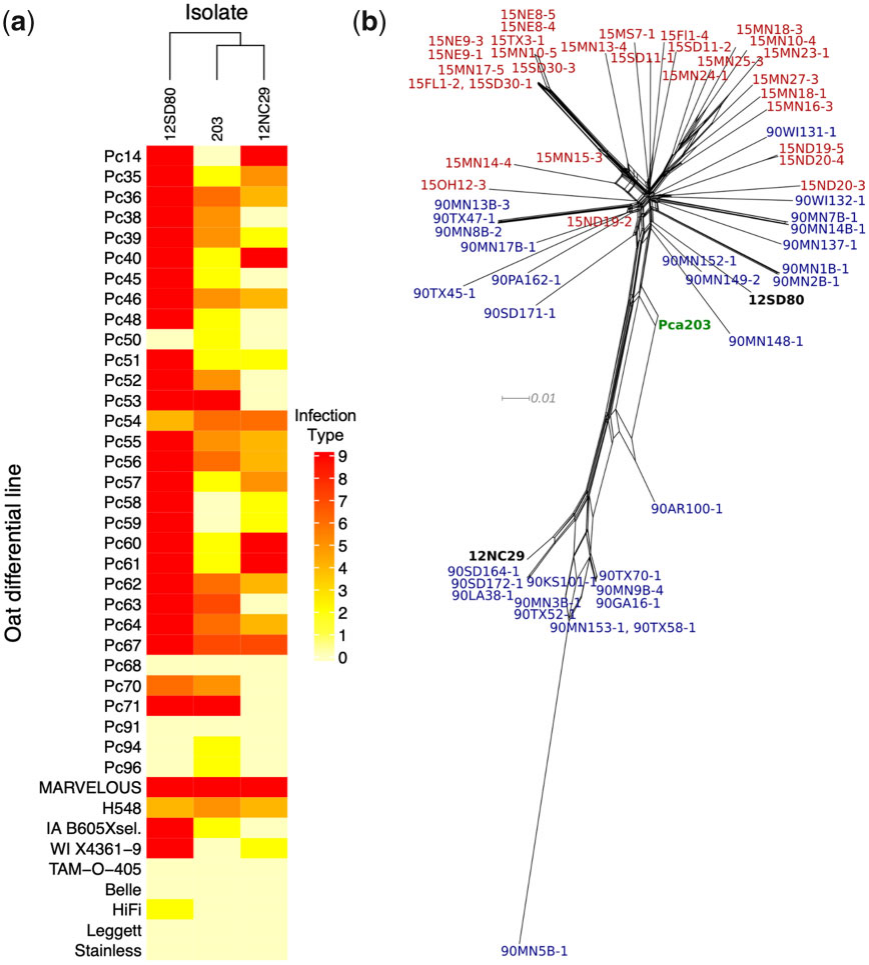
a) Heatmap of linearized rust scores for *Puccinia coronata* f. sp*. avenae* isolates *Pca*203, 12NC29, and 12SD80 on the North American differential set. Infection scores were converted to a numeric scale from 0 (fully resistant) to 9 (fully susceptible) and color coded on a yellow (=0) to red (=9) gradient as shown (side bar) for heatmap generation. Dendrogram (*x*-axis) show hierarchical clustering of isolates with similar virulence patterns. Oat differential lines are shown in the *y*-axis. b) Neighbor-net network (SplitsTree) of *Pca*203, 12SD80, 12NC29, and additional 60 isolates published previously by Miller *et al.* (2020). *Pca* isolates derived from 2015 are shown in red whereas isolates collected in 1990 are shown in blue. *Pca*203 is shown in green, and 12NC29 and 12SD80 are displayed in black.

To create a chromosome-level nuclear phased assembly, we selected an isolate representing the historical race 203 of the oat crown rust pathogen, *Pca*203. The *Pca* race 203 was very common in North America in the 1940s and was still present in the 1970s (Stoa and Swallers 1950; Fleischmann 1967; Fleischmann and Baker 1971). The isolate *Pca*203 was previously revived from a long-term storage collection at the USDA-ARS CDL (Omidvar *et al.* 2018). Here, we reanalyzed the virulence profile and adopted the current pathotype nomenclature to characterize the isolate *Pca*203 stocks of urediniospores. A full report of infection scores of *Pca*203 with the current expanded oat differential set that is currently used in North America (Nazareno *et al.* 2018) was generated (Fig. 1a) and compared with previously sequenced isolates (Miller *et al.*^2018^, ^2020^). *Pca*203 was assigned to pathotype BBBGBCGLLB. Compared with the *Pca* isolates (12NC29 and 12SD80) for which genome references have been previously constructed (Miller *et al.* 2018), *Pca*203 has a unique virulence profile as most *R* genes confer resistance against it (Fig. 1). This suggests that *Pca*203 contains Avr effectors that are absent in 12NC29 and 12SD80 and the *Pca*203 genome can therefore assist in effector identification and studies of virulence evolution for resistance genes represented in the oat differential set. A full comparison to the virulence profiles of isolates derived from 2015 and 1990 (Miller *et al.* 2020) is shown in Supplementary Fig. 1 to demonstrate the abundance of avirulence traits in *Pca*203.

To confirm isolate purity Illumina DNA reads (Supplementary Table 1) from *Pca*203 were mapped to the 12SD80 genome reference (Miller *et al.* 2018), and the allele balance of bi-allelic SNPs was analyzed according to standard methods (Yoshida *et al.* 2013; Li *et al.* 2019). Results showed the expected binary distribution typical of the presence of 2 genomes and thus confirmed the purity of the isolate preparation and absence on contamination with additional unrelated *Pca* genotypes that have been used recently in the laboratory (Supplementary Fig. 2). These data were combined with SNP data from previously sequenced isolates collected in 1990 and 2015 and a neighbor-net network analysis (SplitsTree) derived from 974,924 variant sites to understand the genetic relationship of *Pca203* to these temporally distant *Pca* populations. This analysis placed *Pca203* in a central position between the 2 major clades detected among populations (Fig. 1b). This finding is consistent with *Pca*203 being part of the ancestral North American *Pca* population from which these modern populations are derived, although this is difficult to determine due to the lack of historical population samples spanning years prior 1990.

### Genome assembly, curation, and construction of chromosomes

In total, 51 Gb of PacBio data (∼100× coverage of the *Pca* genome) (Supplementary Table 1) were assembled and polished with 19 Gb of Illumina short reads (∼58× coverage of the *Pca* genome). After removal of contaminant and low coverage contigs this resulted in an assembly of 658 contigs adding to a total size of 206.4 Mb (Supplementary Table 2). Given that the haploid genome assembly size of *Pca* isolates 12SD80 and 12NC29 were estimated at 99.2 and 105.3 Mb, respectively (Miller *et al.* 2018), these results suggest that the 2 haplotypes were captured by this approach. Collapsed regions were determined by inspecting the average PacBio read coverage in 1,000 bp bins (Supplementary Fig. 3). The first large peak at ∼177× coverage represents the coverage for most of the haploid or noncollapsed regions. A second small peak near 234x coverage, or double the haploid coverage, likely represents collapsed regions. A total of 1.8 Mb of sequence showed coverage >175x (>1.5 times haploid coverage). Thus, the *Pca*203 assembly only has low numbers of collapsed regions.

**Fig. 2.**
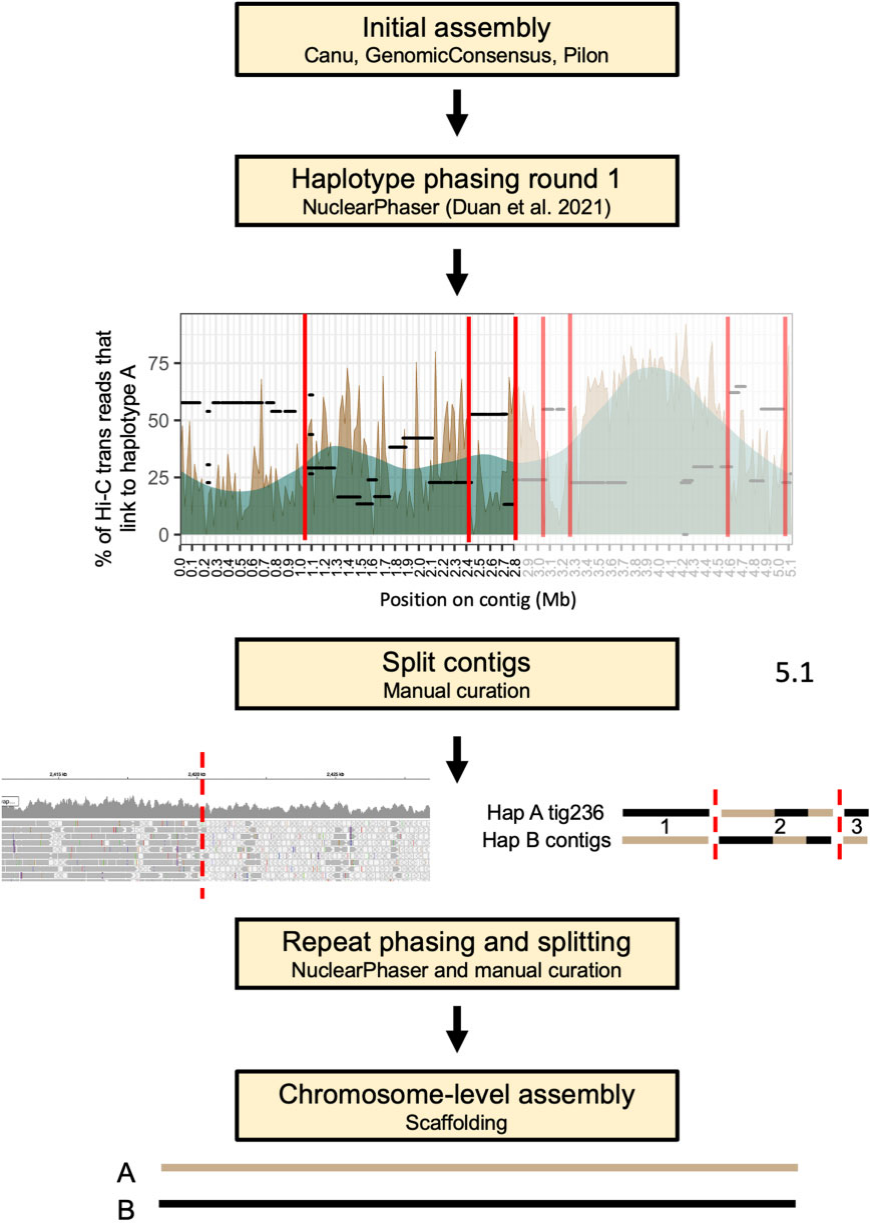
Flowchart illustrating the key steps in the haplotype phasing and chromosome level assembly of *Pca*203. The NuclearPhaser pipeline (Duan *et al.* 2022) was used to create a fully phased, chromosome-level assembly. First, NuclearPhaser constructs a highly confident subset of the 2 haplotypes that are expected to reside in separate nuclei and identifies potential phase swaps in the 2 preliminary haplotype sets. A high proportion of *trans* Hi-C reads should map within the A haplotype, so positions where the proportion drops are flagged as suspect for phase swaps. We inspected these potential phase switch breakpoints using Illumina read mappings. As shown in the figure, a change to high coverage multimapping reads indicates high similarity between regions across haplotypes that may have resulted in a phase swap. After correcting phase swaps, the NuclearPhaser pipeline was used again with the updated genome. Lastly, the 2 haplotypes were scaffolded separately with Hi-C data into chromosomes.

**Fig. 3.**
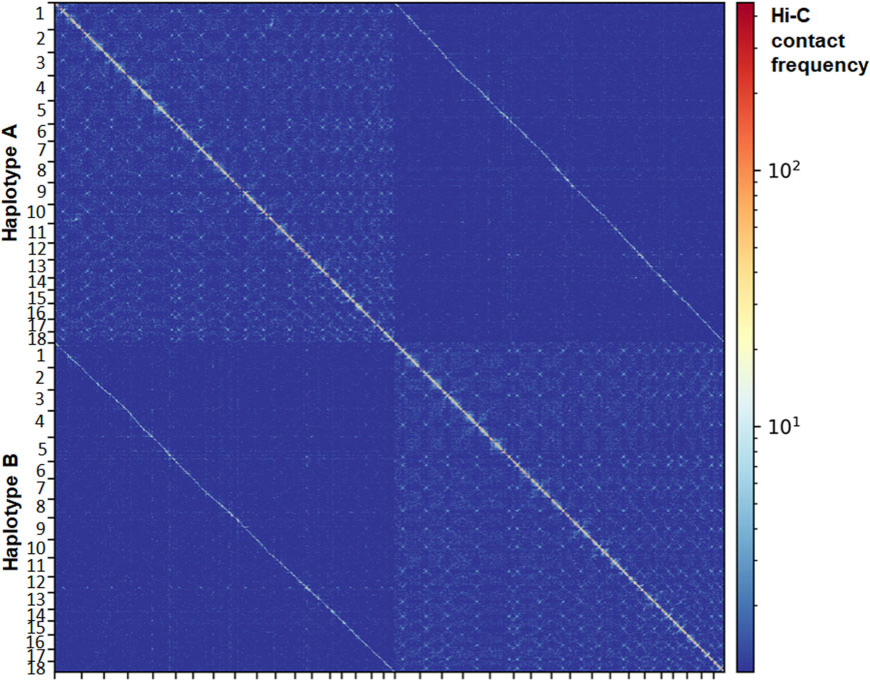
Hi-C contact map for the *Puccinia coronata* f. sp. *avenae* isolate *Pca*203 A and B haplotypes in 100 kb resolution. The 2 haplotypes exhibit a clear phasing signal, with spurious Hi-C contacts between the haplotypes visible as a weak additional diagonal line in the upper right and lower left corners. The 2*18 centromeres are visible as bowtie-shapes in the contact maps. Color scale corresponds to the number of contacts in each 100 kb bin ranging from blue (0 contacts) through to red (>1,000 contacts) as indicated in the right hand scale.

**Fig. 4.**
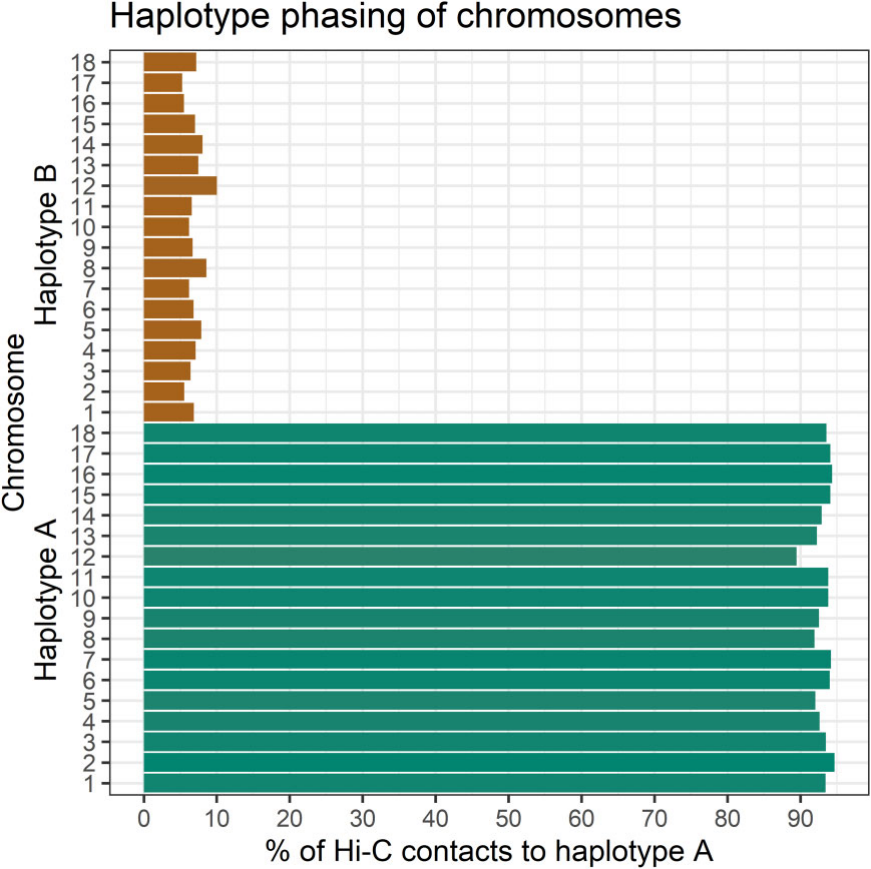
Hi-C cis and *trans* contact distribution relative to haplotype A. The haplotype A chromosomes have on average over 90% of their Hi-C links to haplotype A, whereas the haplotype B chromosomes have <10% of their Hi-C links to haplotype A.

The initial *Pca*203 assembly was phased using the NuclearPhaser pipeline (Duan *et al.* 2022), which uses a Hi-C graph approach to phase dikaryon assemblies and includes a step for identifying phase swaps (Fig. 2). As a first step NuclearPhaser was used to preliminarily assign contigs to the 2 haplotypes and identify phase swaps using Hi-C contact information. We identified phase swaps in 30 contigs, which were manually corrected by breaking these contigs at the phase switch sites. NuclearPhaser was then used a second time and an additional 7 breakpoints were identified in 3 contigs. After phase correction, the 2 haplotypes were scaffolded separately into chromosomes using the Hi-C data. Scaffolding resulted in 18 chromosomes in each haplotype, covering 101.696 and 98.492 Mb, with 173 small contigs covering 7.973 Mb remaining unassigned to either haplotype (Table 1). Hi-C contact maps were then inspected to identify centromeres in each chromosome of both haplotypes. All centromeres were visible on the Hi-C contact maps for each of the 2*18 chromosome (Fig. 3), which were ordered according to the numbering of the homologous chromosomes in *Puccinia graminis* f. sp. *tritici* (Li *et al.* 2019). Correct haplotype phasing was confirmed by evaluating the distribution of Hi-C contacts to haplotype A (Fig. 4). Telomere sequence analysis identified 48 total telomeres of the expected 72, with 20 of 36 in haplotype A and 28 of 36 in haplotype B. The high number of identified telomeres suggest that the entire sequence was acquired for most chromosomes.

**Table 1.** Genome assembly and annotation statistics for the chromosome-level nuclear phased genome reference of *Puccinia coronata* f. sp. *avenae* isolate *Pca*203.

	203 Haplotype A	203 Haplotype B	Unassigned*a*
Total size (Mbp)	101.696	98.492	7.973
Number of chromosomes	18	18	173 contigs
Number of telomeres	20	28	7
BUSCOs for the combined assembly			
Complete		93.4%	
Single-copy		9.7%	
Duplicated		83.7%	
Fragmented		2.9%	
Missing		3.7%	
Annotated genes	17,877	17,294	1,322
Gene content of genome (%)	25.0	25.2	20.3
Number of repeats identified	146,879	141,763	10,448
Repeat content of genome (%)	56.3	56.2	60.9
Secreted proteins	1,985	1,943	108
Predicted effectors	1,005	972	52

a Unassigned contigs were not placed in either haplotype.

**Fig. 5.**
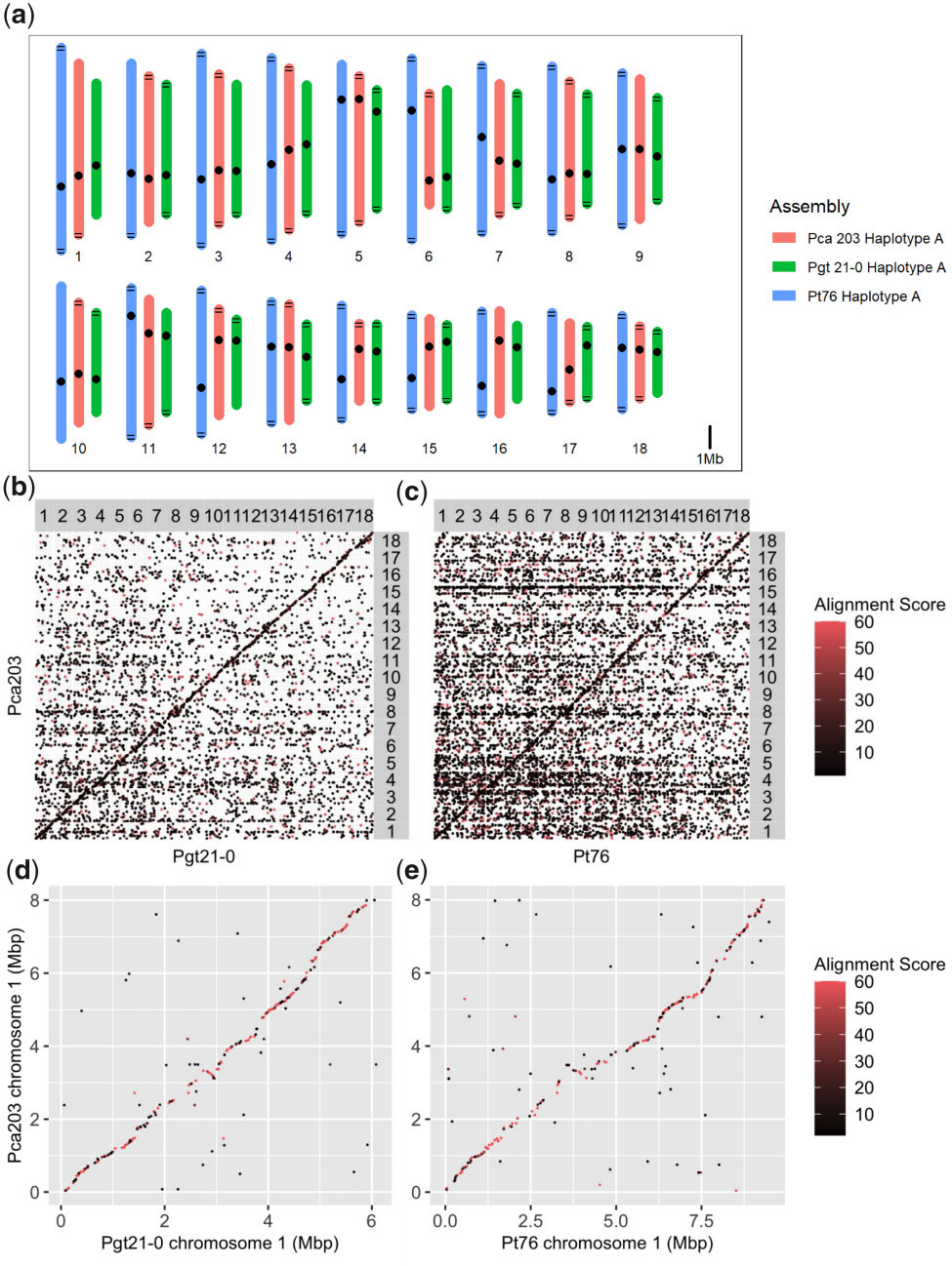
a) Chromosome size comparison between *Puccinia coronata* f. sp. *avenae* isolate *Pca*203 (red), *Puccinia graminis* f. sp. *tritici* isolate *Pgt*21-0 (green), and *Puccinia triticina* isolate *Pt*76 (blue), with dots representing centromeres and black horizontal lines representing identified telomeres. Dotplot alignment between: b) the isolate *Pca*203 A genome and isolate *Pgt*21-0 A genome c) isolate *Pca*203 A genome and isolate *Pt*76 A genome d) isolate *Pca*203 chromosome 1A and isolate *Pgt*21-0 chromosome 1A e) isolate *Pca*203 chromosome 1A and isolate *Pt*76 chromosome 1A. Alignment scores are the mapQ values produced by minimap2; points with MAPQ = 0 are excluded to remove multimapping.

The completion of the *Pca*203 genome assembly provides the opportunity for comparison to the preexisting high quality references for wheat stem rust (*Pgt*21-0) and wheat leaf rust (*Pt*76). Like the chromosome-level nuclear phased assemblies for *Pgt*21-0 and *Pt*76, the *Pca*203 assembly has 18 chromosome pairs (Fig. 4). Sequence identity between *Pca*203 and both *Pgt* 21-0 and *Pt*76 is relatively low, as illustrated by dotplot alignments (Fig. 5). Genome annotation using RNAseq data from *Pca*203, 12SD80, and 12NC29 yielded a total of 36,493 genes, with 17,877 on haplotype A, 17,294 on haplotype B, and the remaining 1,322 on unassigned scaffolds (Table 1). Gene space occupies 25.0% on the A haplotype, 25.2% on the B haplotype, and 20.3% of unassigned scaffolds (Table 1). In comparison, repeat sequences represent 56.5% of the entire genome, with slightly greater prevalence on the unassigned scaffolds compared with the chromosomes (Table 1). Of the 36,524 annotated genes, 4,036 were identified as secreted proteins, and 2,029 were predicted as effectors by EffectorP 3.0 (Table 1). Orthogroups were determined from the annotation information from haplotypes A and B; in haplotype A, 91.6% (16,382) of genes were assigned to orthogroups while in haplotype B this was 92.2% (15,952).

**Fig. 6.**
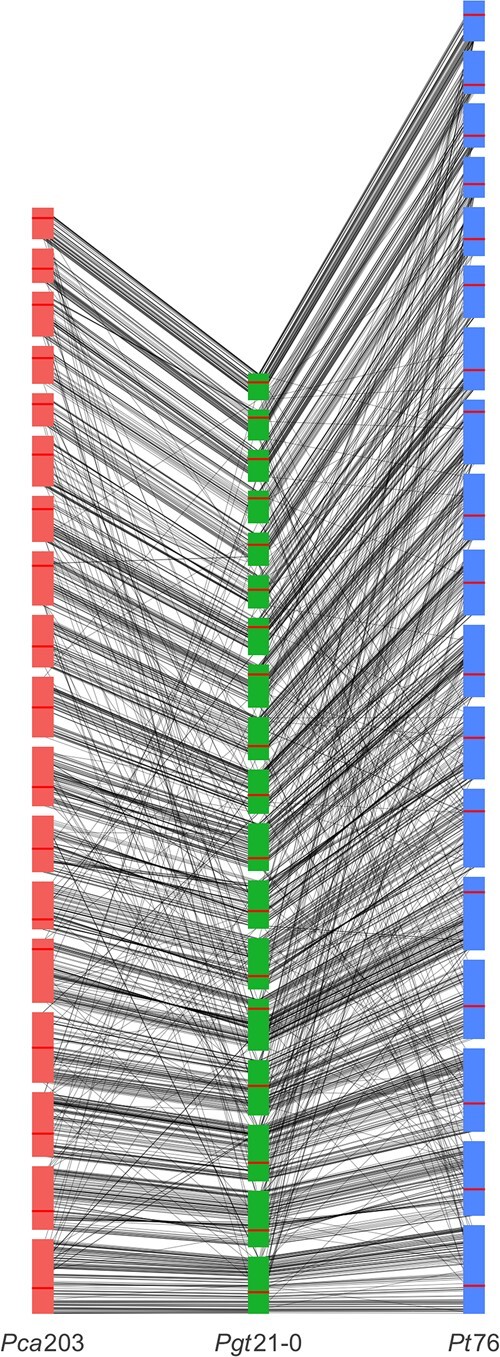
Synteny plot demonstrating connections between BUSCO genes of the *Puccinia coronata* f. sp. *avenae* isolate *Pca*203 haplotype A chromosomes (left) and the haplotype A chromosomes of the cereal rust species *P. graminis* f. sp. t*ritici* (*Pgt*21-0, center) and *P. triticina* (*Pt76*, right). Horizontal red lines in chromosomes indicate position of the centromere. Chromosomes are ordered from 1 to 18 ascending from bottom.

Interestingly, gene synteny among all 3 cereal rust species is relatively high, as illustrated by the positions of the BUSCO genes shown in Fig. 6. Compared with *Pgt* 21-0 and *Pt*76, *Pca*203 has slightly more annotated genes than *Pt*76 (31,930) and slightly fewer than *Pgt* 21-0 (38,007). The proportion of the *Pca*203 genome covered by genes is ∼25% in both the A and B haplotypes, with unplaced contigs being slightly less gene-rich (20%). The gene space in *Pca*203 is comparable to *Pt*76 (24.6%) and lower than that of *Pgt*21-0 (34.9%).

## Discussion

So far, only a few genome references for rust fungi have been assembled to fully represent phased haplotypes and chromosome sequences. These include the *Pgt*21-0 isolate of the stem rust fungus *P. graminis* f. sp. *tritici* (Li *et al.* 2019) and the *Pt*76 isolate of the leaf rust fungus *P. triticina* (Duan *et al.* 2022). Our study introduces the third rust species for which a chromosome-level nuclear phased assembly is available, namely the oat crown rust pathogen, *P. coronata* f. sp. *avenae* (*Pca*). These 3 *Puccinia* species have 2*18 chromosomes that display high gene synteny despite low overall sequence identity, thus reflecting the close evolutionary history of the species (Aime *et al.* 2018; Aime and McTaggart 2021).

The chromosome level and nuclear phased assembly of *Pca*203 will enable future pathogenicity studies of this important oat pathogen. At present, no effector gene has been identified for *Pca* and no *R* genes have been isolated from oat or its wild relatives, which is a major bottleneck for disease management strategies. We foresee that access to a gold standard genome reference for *Pca* will accelerate progress in understanding the molecular and genetic basis of the oat crown rust pathosystem. From a plant pathology perspective, the race 203 of *Pca* offers the opportunity to unravel a puzzling question in the field, namely the relationship between genetic resistance to biotrophic pathogens and susceptibility to necrotrophic pathogens. The deployment and widespread use of Victoria and Victoria related oats in the USA as response to oat crown rust epidemics likely caused by *Pca* predominant races such as 203 resulted in significant Victoria Blight epidemics between 1946 and 1948 (Lorang *et al.* 2007). The fungus *C. victoriae*, causal agent of Victoria Blight, produces a toxin called victorin which is crucial for pathogenicity of *C. victoriae*. Both toxin sensitivity and Victoria blight disease susceptibility are conferred by the gene named *Vb* (Wolpert *et al.* 2002). The *Vb* gene appears to be genetically linked to the resistance to race 203, conferred by the *Pc2* gene, and it has been suggested that both traits are controlled by the same gene (Welsh *et al.* 1954; Mayama *et al.* 1995; Wolpert *et al.* 2002). The effector that would be recognized by *Pc2*, *AvrPc2*, has not been identified yet; however, the *Pca*203 genome assembly may aid in the identification as the *AvrPc2* sequence should be represented in the assembly. Given the extensive characterization of Victorin and additional genetic components (Wolpert *et al.* 1985; Wolpert and Macko 1989; Lorang *et al.* 2007; Kessler *et al.* 2020) dictating the outcome of this plant pathogen interaction, the identification of *AvrPc2* is an important piece to understand the tradeoffs of genetic resistance. As highlighted by the virulence profile of *Pca*203, other effectors recognized by dozens of immunoreceptors in oat should also be present in this assembly.

In the past few decades, *Pca* has drastically shifted toward a wider spectrum of virulence (Miller *et al.* 2020). While this situation was analyzed in depth within US-derived populations, researchers from all over the world have made similar observations. Thus, the development of virulence markers and robust surveillance activities with capacity for large numbers of samples seems particularly critical for pathosystems like oat crown rust which display rapid pathogen evolution and need for durable genetic resistance. This genome reference will aid in the discovery of effector allelic variants, which will enable researchers to develop such tools for virulence monitoring strategies.

## Data availability

Sequencing reads, the assembly and annotation are available at the CSIRO Data Portal https://data.csiro.au/collection/csiro:53477. Scripts for identification of contaminants, collapsed regions, and telomeres are available at https://github.com/JanaSperschneider/GenomeAssemblyTools and https://github.com/JanaSperschneider/FindTelomeres. NuclearPhaser is available at https://github.com/JanaSperschneider/NuclearPhaser. Scripts and data used to construct figures are available at https://github.com/henni164/Pca203_assembly. Sequencing data from the SRA numbers listed in Supplementary Table 1.

Supplemental material is available at *G3* online.

## Supplementary Material

jkac149_Supplementary_Figure_S1

jkac149_Supplementary_Figure_S2

jkac149_Supplementary_Figure_S3

jkac149_Supplemental_Material_Table_S1

jkac149_Supplemental_Material_Table_S2

jkac149_Supplemental_Material_Legends
