# Supplementary figures and images for: A chromosome-level, fully phased genome assembly of the oat crown rust fungus Puccinia coronata f. sp. avenae: a resource to enable comparative genomics in the cereal rusts
Source: G3 (Bethesda). 2022 Jun 22;12(8):jkac149. doi: 10.1093/g3journal/jkac149 (PMC9339303; doi:10.1093/g3journal/jkac149)

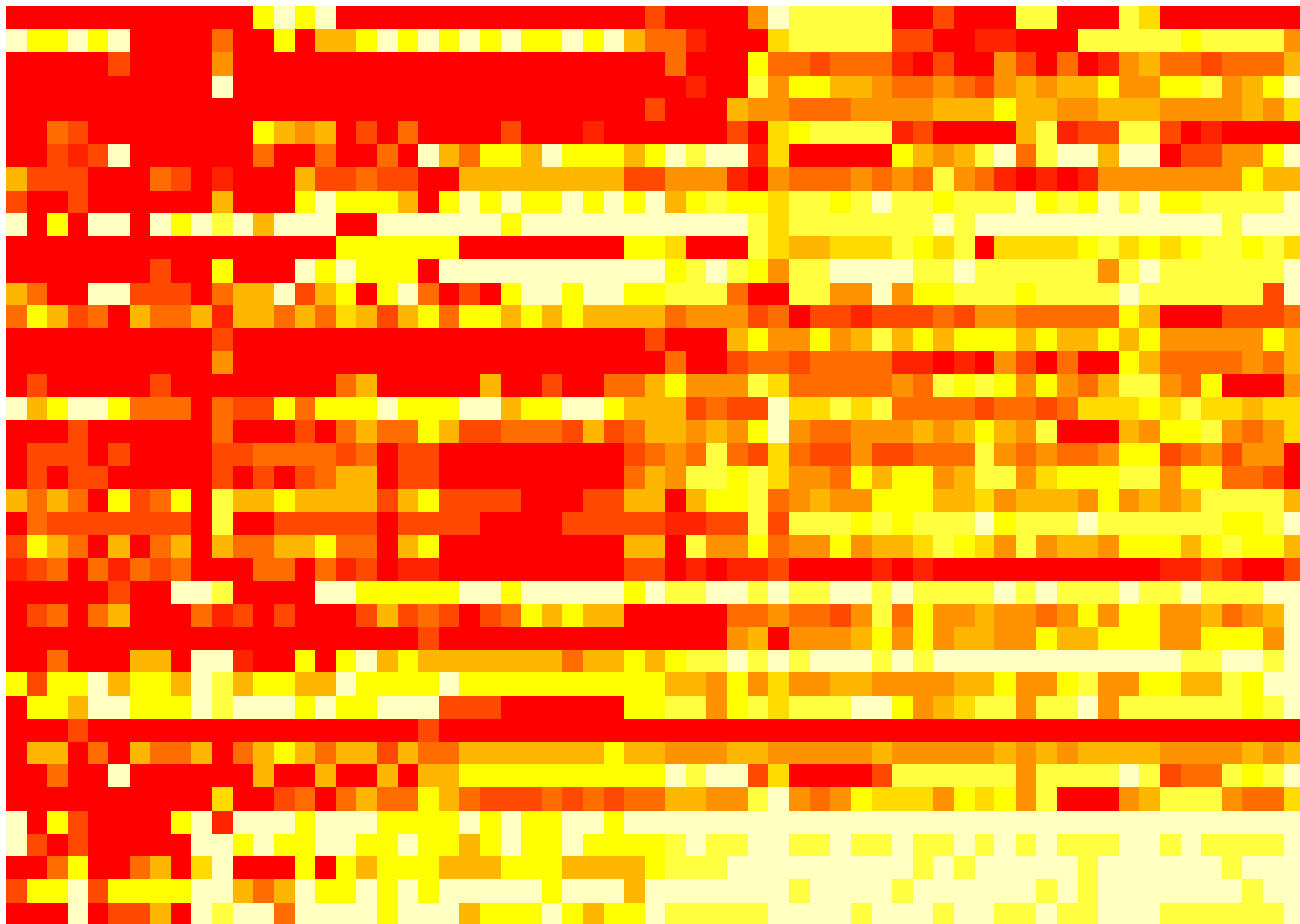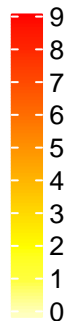

Supplement: jkac149_Supplementary_Figure_S1 [file jkac149_supplementary_figure_s1.pdf]

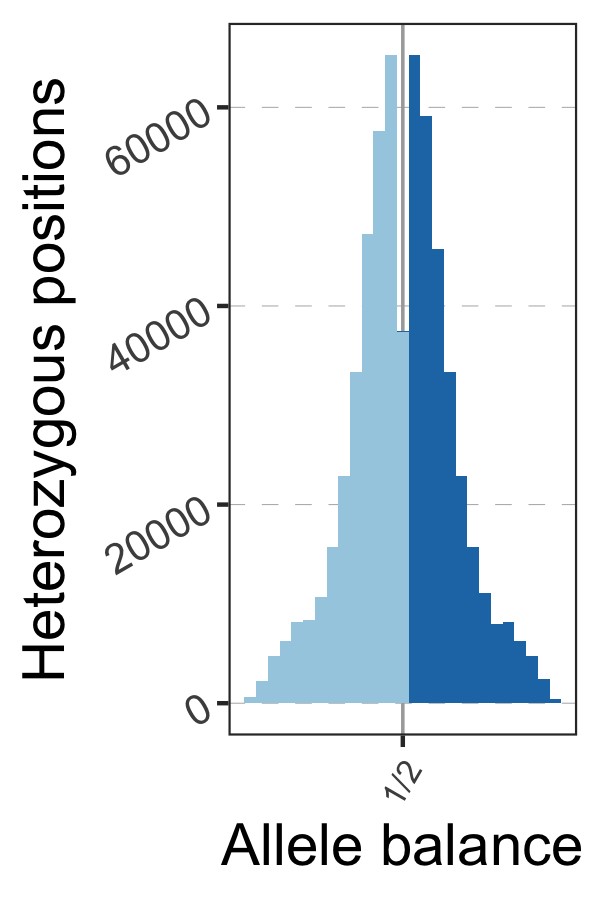

Supplement: jkac149_Supplementary_Figure_S2 [file jkac149_supplementary_figure_s2.jpeg]

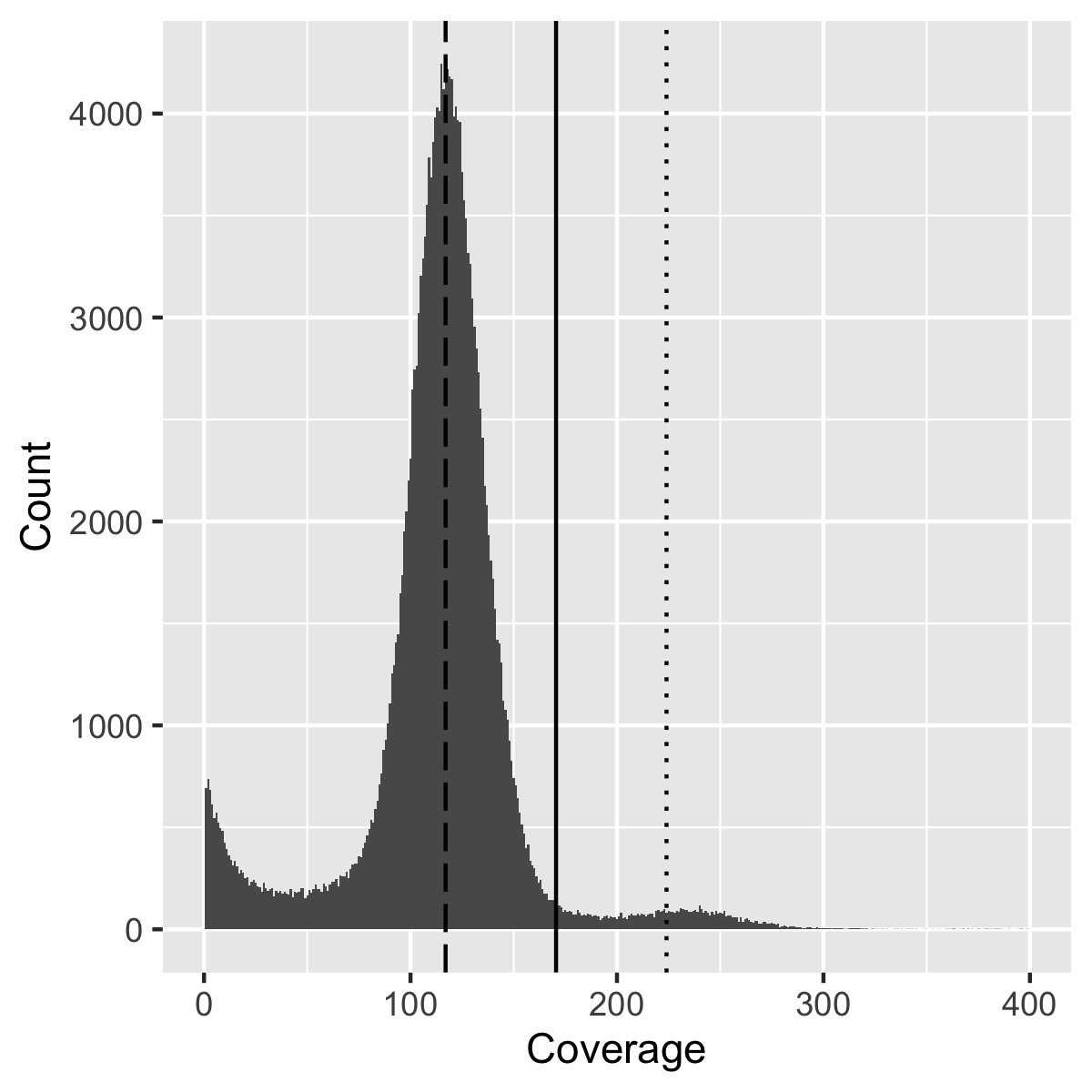

Supplement: jkac149_Supplementary_Figure_S3 [file jkac149_supplementary_figure_s3.jpeg]
