# Supplementary material for: A chromosome-level, fully phased genome assembly of the oat crown rust fungus Puccinia coronata f. sp. avenae: a resource to enable comparative genomics in the cereal rusts
Source: G3 (Bethesda). 2022 Jun 22;12(8):jkac149. doi: 10.1093/g3journal/jkac149 (PMC9339303; doi:10.1093/g3journal/jkac149)
Supplement: jkac149_Supplemental_Material_Table_S1 [file jkac149_supplemental_material_table_s1.docx]

**Table S1.** Sequencing statistics for raw reads used in the *Puccinia coronata* f. sp. *avenae* isolate *Pca*203 genome assembly and annotation.

| SRA | Type | Sequencer | Total Raw Reads | Total length of reads (bp) | Average coverage |
| --- | --- | --- | --- | --- | --- |
|  | Genomic DNA | Pacbio Sequel II | 9,041,864 | 28,267,975,258 | 135.8 |
|  | Genomic DNA | Illumina NovaSeq S2 | 50,934,284 | 7,691,076,884 | 36.9 |
|  | RNA (2dpi) | Illumina NextSeq | 149,868,264 | 11,389,988,064 | - |
|  | RNA (5dpi) | Illumina NextSeq | 161,754,544 | 12,293,345,344 | - |
|  | Hi-C Library | Illumina | 130,793,620 | 19,619,043,000 | 94.2 |
