# Supplementary material for: A chromosome-level, fully phased genome assembly of the oat crown rust fungus Puccinia coronata f. sp. avenae: a resource to enable comparative genomics in the cereal rusts
Source: G3 (Bethesda). 2022 Jun 22;12(8):jkac149. doi: 10.1093/g3journal/jkac149 (PMC9339303; doi:10.1093/g3journal/jkac149)
Supplement: jkac149_Supplemental_Material_Table_S2 [file jkac149_supplemental_material_table_s2.docx]

**Table S2.** Genome assembly statistics for *Puccinia coronata* f. sp. avenae isolate *Pca*203 after polishing and removal of contaminants and mitochondrial sequences and before scaffolding and haplotype phasing. Genome assembly statistics of 12SD80 and 12NC29 were included for comparison purposes.

|  | **Rust isolate assembly** | | | | |
| --- | --- | --- | --- | --- | --- |
|  | *Pca*203 | 12SD80 primary* | 12SD80 haplotigs* | 12NC29 primary* | 12NC29 haplotigs* |
| Total # contigs | 658 | 603 | 1033 | 777 | 950 |
| Largest contig (bp) | 5,222,753 | 1,390,849 | 353,514 | 1,189,025 | 483,040 |
| Total length (bp) | 206,392,043 | 99,159,039 | 51,308,767 | 105,248,506 | 61,031,715 |
| L50 (bp) | 798,317 | 268,263 | 77,751 | 217,305 | 121,163 |
| N50 | 53 | 118 | 207 | 146 | 160 |
| GC (%) | 44.65 | 44.67 | 44.91 | 44.66 | 44.88 |

* For the 12SD80 and 12NC29 genome assemblies, data represents the current state of the published references (Miller et al. 2020)
