# Supplementary material for: A chromosome-level, fully phased genome assembly of the oat crown rust fungus Puccinia coronata f. sp. avenae: a resource to enable comparative genomics in the cereal rusts
Source: G3 (Bethesda). 2022 Jun 22;12(8):jkac149. doi: 10.1093/g3journal/jkac149 (PMC9339303; doi:10.1093/g3journal/jkac149)
Supplement: jkac149_Supplemental_Material_Legends [file jkac149_supplemental_material_legends.docx]

**Table S1.** Sequencing statistics for raw reads used in the *Puccinia coronata* f. sp. *avenae* isolate *Pca*203 genome assembly and annotation.

**Table S2.** Genome assembly statistics for *Puccinia coronata* f. sp. avenae isolate *Pca*203 after polishing and removal of contaminants and mitochondrial sequences and before scaffolding and haplotype phasing. Genome assembly statistics of 12SD80 and 12NC29 were included for comparison purposes.

**Figure S1**. Heatmap of linearized rust scores on the North American differential set for *Puccinia coronata* f. sp. *avenae* isolates *Pca*203, 12SD80, 12NC29, 30 isolates from 1990 and 30 isolates from 2015 as previously published in Miller et al. (2020). Infection scores were converted to a numeric scale (0 = resistance shown in yellow to 9 = susceptibility shown in red) for heatmap generation. Dendrogram (x-axis) shows hierarchical clustering of isolates with similar virulence patterns. Oat differential lines are shown in the y-axis.

**Figure S2**. Allele balance plot of variants in *Puccinia coronata* f. sp. *avenae* isolate *Pca*203 when mapped against the 12SD80 reference genome.

**Figure S3**. Histogram of the average coverage in 1000 bp bins across the cleaned *Puccinia coronata* f. sp. *avenae* isolate *Pca*203 genome assembly. Dashed line (x = 117) represents the coverage mode, which represents the haploid coverage for the assembly. The dotted line represents double the haploid coverage (x = 234). The solid line (x = 175) is the cutoff used for determining whether a region was considered collapsed.
